# CNN4Essential: a convolutional neural network model for predicting bacterial gene essentiality based on multi-feature fusion

**DOI:** 10.1186/s12864-026-12819-3

**Published:** 2026-04-06

**Authors:** Yuan-Nong Ye, Ren-Yu Zhou, Lan-Yang Li, Hua-Ting Yuan, Jie Xia, Ya-Wei Li, Zhu Zeng, Xiao-Ya Zhang

**Affiliations:** 1 Cells and Antibody Engineering Research Center of Guizhou Province, Key Laboratory of Biology and Medical Engineering, School of Biology and Engineering, Guizhou Medical University, Guiyang, 550025 China; 2 Bioinformatics and Biomedical Big Data Mining Laboratory, Department of Medical Informatics, Guizhou Medical University, Guiyang, 550025 China

**Keywords:** Essential genes, Multi-feature, Convolutional neural network, Gene prediction, Data fusion

## Abstract

**Background:**

Accurately identifying essential genes in bacteria is critical for understanding microbial biology and developing novel antibiotics. However, the heterogeneity of biological data poses a challenge for reliable prediction. This study aims to enhance prediction accuracy by integrating diverse biological features through a multi-feature fusion framework.

**Results:**

This study combined sequence data, gene annotations, protein–protein interaction networks, and subcellular localization information to construct a convolutional neural network (CNN)-based model, CNN4Essential. Feature importance was assessed using a random forest algorithm, and dimensionality reduction was performed with truncated singular value decomposition. The model was evaluated through intra-species prediction (INSP) and leave-a-species-out prediction (LASP) across 22 prokaryotic species. CNN4Essential achieved an average AUC of 0.884 in INSP, 0.726 in LASP, and 0.851 across all species, outperforming existing methods. Furthermore, predictions for *Haemophilus influenzae* were compared with known drug-target genes from DrugBank. A positive correlation between prediction scores and target gene matching rates was observed.

**Conclusions:**

The integration of multi-source features with a deep learning model significantly improves bacterial essential gene prediction. CNN4Essential not only surpasses single-feature and shallow models in performance but also holds promise for identifying potential drug targets.

**Supplementary Information:**

The online version contains supplementary material available at 10.1186/s12864-026-12819-3.

## Background

Essential genes are defined as those that are critical for maintaining the vital functions of an organism, particularly under specific environmental conditions [1]. The absence of essential genes generally leads to profound functional impairments, including the inability to replicate, reproduce, or maintain cellular homeostasis, which may ultimately result in cell death [2]. Consequently, the identification of essential genes in bacteria is of considerable importance, particularly in the contexts of drug development [3] and the management of pathogenic microorganisms [4].

Traditionally, essential genes have been identified through experimental methodologies such as gene knockout [5], transposon insertion mutagenesis [6], RNA interference [7], and CRISPR/Cas9 gene editing [8]. These techniques involve the deletion or silencing of target genes followed by the observation of resultant phenotypic effects. While these approaches are effective, they are also labor-intensive, time-consuming, and costly, particularly when applied to the study of numerous genes across diverse species or strains.

Computational methodologies present a highly efficient alternative to traditional experimental techniques by employing bioinformatics tools and algorithms to predict essential genes utilizing omics data. A range of approaches, including sequence-based and network-based methods, are employed in this context [9–12]. Furthermore, machine learning and deep learning techniques are increasingly being utilized to integrate multiple data sources. These computational strategies are crucial for a variety of applications, with a discernible trend towards the adoption of integrated computational approaches [13–16].

The advances in bioinformatics and various omics technologies have led to the explosion of diverse biological data, and big data-based computational methods for identifying essential genes have become a focus of much attention. However, most existing identification methods rely primarily on single-source data, such as gene expression data or protein interaction data, which limits their identification effectiveness [17–19]. Although recognition methods based on integrating genome, transcriptome, and proteome data have greater advantages in theory, the practical application results are not ideal [20]. This is primarily attributed to the high complexity of integrating and analyzing multisource data, as well as the significant heterogeneity that exists among different data sources.

Due to the aforementioned limitations, A multi-source data fusion approach is proposed, which integrates diverse data types from different sources into a unified, structured dataset for analysis. Our method effectively addresses the issue of inadequate fusion of data from various sources in current prediction algorithms. By fully utilizing various experimental data, a model is developed to achieve high-accuracy predictions of bacterial essential genes, overcoming the challenges associated with data heterogeneity and integration complexity in current prediction methods.

With the rapid advancements in bioinformatics, integrating heterogeneous data sources to construct high-precision and generalizable predictive models has become a growing trend. In this context, Our study introduces and systematically evaluates a Convolutional Neural Network (CNN) model, termed CNN4Essential, based on the fusion of four types of heterogeneous data features. The model combines sequence features, protein-protein interaction (PPI) network features, Gene Ontology (GO) annotations, and subcellular localization data to accurately identify bacterial essential genes. CNN4Essential was trained on several bacterial datasets, and its performance was evaluated using intra-species prediction, leave-one-species-out prediction, and all-species prediction methods. The results demonstrate that the model achieves high accuracy, stability, and generalization ability in identifying essential genes.

The primary objective of this research is to improve the feature data quality in computational methods, thereby enhancing the effectiveness of deep learning models for essential gene recognition through multi-source data fusion, providing more reliable tools for biological research and applications.

## Results and analysis

### Performance of intra-species prediction

**Fig. 1:**
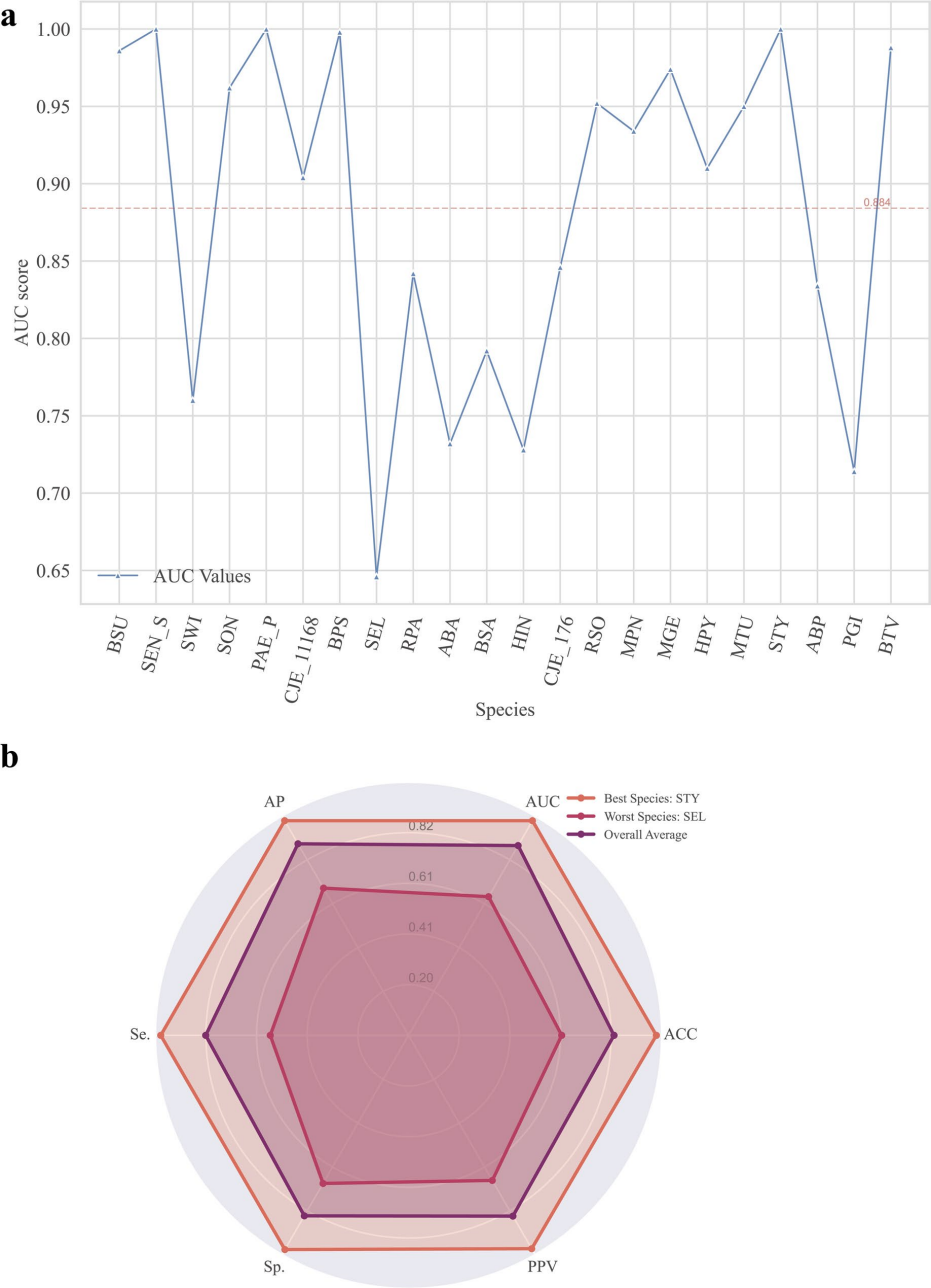
Evaluation of intra-species prediction performance for 22 bacterial species. **a** AUC scores for intra-species prediction are averaged over five iterations. The red dashed line represents the average value of intra-species predictions for 22 species. **b** Scores for the performance metrics for intra-species prediction are averaged over five iterations. AP, average accuracy; AUC, area under the receiver operating characteristic curve; Se., Sp., and ACC, sensitivity, specificity, and accuracy, respectively; PPV, positive predictive value

In intra-species prediction (INSP), both the training and test data are derived from the same species. Six performance metrics were calculated using 10-fold cross-validation repeated five times. The AUC values across species ranged from 0.646 to 1.000, with an average of 0.884. The AUC values for each bacterial species are shown in Table 1 and Fig. 1a. Other performance metrics were as follows: accuracy 82.9%, average precision 89.2%, sensitivity 81.9%, specificity 84.1%, and positive predictive value 84.2%. Among the tested species, Salmonella typhimurium LT2 (STY) exhibited the best performance, while Synechococcus elongatus PCC 7942 (SEL) showed the poorest performance (Fig. 1b).

**Table 1:** AUC scores for intra-species and leave-a-species-out predictions in 22 prokaryotic species

No.	Species	Abbr.	Intra-species predictions	Leave-a-species-out predictions
1	*Bacillus subtilis* 168	BSU	0.986	0.875
2	*Haemophilus influenzae* Rd KW20	HIN	0.728	0.568 *
3	*Mycoplasma genitalium* G37	MGE	0.974	0.598 *
4	*Helicobacter pylori* 26,695	HPY	0.91	0.592 *
5	*Mycobacterium tuberculosis* H37Rv	MTU	0.95	0.659
6	*Salmonella typhimurium* LT2	STY	1	0.875
7	*Acinetobacter baylyi* ADP1	ABP	0.834	0.685
8	*Porphyromonas gingivalis* ATCC 33,277	PGI	0.714	0.712
9	*Bacteroides thetaiotaomicron* VPI-5482	BTV	0.988	0.779
10	*Salmonella enterica* subsp. Enterica serovar Typhimurium str. 14,028 S	SEN_S	1	0.769
11	*Sphingomonas wittichii* RW1	SWI	0.76	0.615
12	*Shewanella oneidensis* MR-1	SON	0.962	0.817
13	*Pseudomonas aeruginosa* PAO1	PAE_P	1	0.611
14	*Campylobacter jejuni* subsp. Jejuni NCTC 11,168 = ATCC 700,819	CJE_11168	0.904	0.848
15	*Burkholderia pseudomallei* K96243	BPS	0.998	0.797
16	*Synechococcus elongatus* PCC 7942	SEL	0.646	0.632
17	*Rhodopseudomonas palustris* CGA009	RPA	0.842	0.713
18	*Acinetobacter baumannii* ATCC 17,978	ABA	0.732	0.699
19	*Brevundimonas subvibrioides* ATCC 15,264	BSA	0.792	0.716
20	*Campylobacter jejuni* subsp. Jejuni 81–176	CJE_176	0.846	0.653
21	*Ralstonia solanacearum* GMI1000	RSO	0.952	0.728
22	*Mycoplasma pneumoniae*	MPN	0.934	0.618
	Mean AUC score		0.884	0.707

^(*)^ Indicates species with AUC score < 0.6 by the leave-a-species-out prediction

### Performance evaluation of leave-a-species-out prediction

**Fig. 2:**
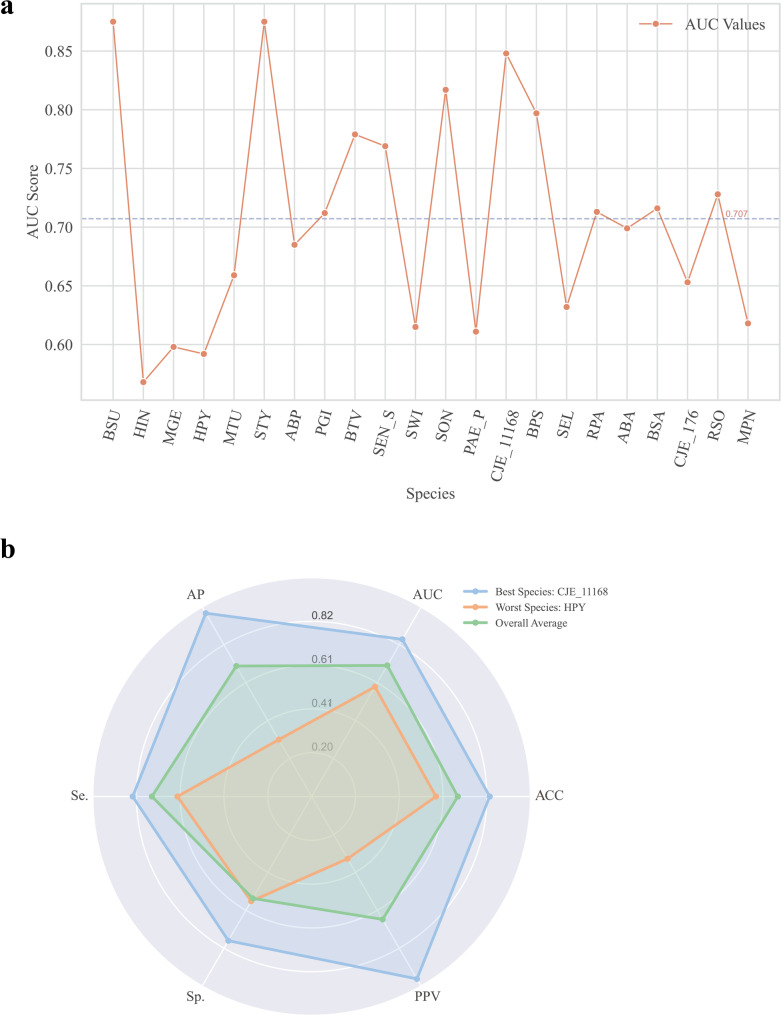
Evaluation of leave-a-species-out prediction performance for 22 bacterial species. **a** AUC scores for leave-a-species-out prediction are averaged over five iterations. **b** Scores for the performance metrics for leave-a-species-out prediction are averaged over five iterations. AP, average accuracy; AUC, area under the receiver operating characteristic curve; Se., Sp., and ACC, sensitivity, specificity, and accuracy, respectively; PPV, positive predictive value

In the leave-a-species-out prediction (LASP) setting, one species was reserved as the test set, while the others were used for training. After performing ten-fold cross-validation and retaining the best model for LASP, the AUC values for each species were 0.568–0.875, with *Campylobacter jejuni* (CJE_11168) achieving the highest score and *Helicobacter pylori* 26,695 showing lower performance (Table 1, Fig. 2a and b).

### CNN4Essential model comparison in INSP, LASP and all-species prediction

#### CNN4Essential model outperforms traditional machine learning methods in INSP

The CNN4Essential model, trained on a multi-feature dataset, outperformed traditional machine learning methods in INSP. It achieved an average AUC of 0.884, surpassing other models: Nigatu et al. (AUC 0.84) [21], Liu et al. (AUC 0.791) [15] and Xu et al. (AUC 0.728) [22]. Detailed comparisons are presented in Table 2.

**Table 2:** Comparing the AUC score of INSP with other models

	AUC value	Main features	Model
CNN4Essential	0.884	Fusion of four types of features	CNN
Nigatu et al. (2017) [21]	0.84	Features of information theory	RF
Liu et al. (2021) [15]	0.791	Features of information theory	BPNN
Xu et al. (2019) [22]	0.728	Features of 57 sequence	ANN

#### CNN4Essential model in LASP demonstrates strong generalization ability and robustness

**Fig. 3:**
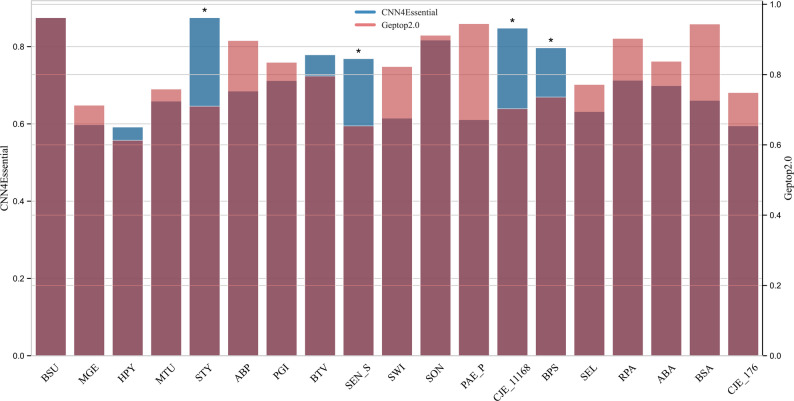
Dual-axis comparison of CNN4Essential and the Geptop2.0 results for the same species. Asterisk (*) indicate species for which CNN4Essential had higher AUC values than those for Geptop2.0

**Fig. 4:**
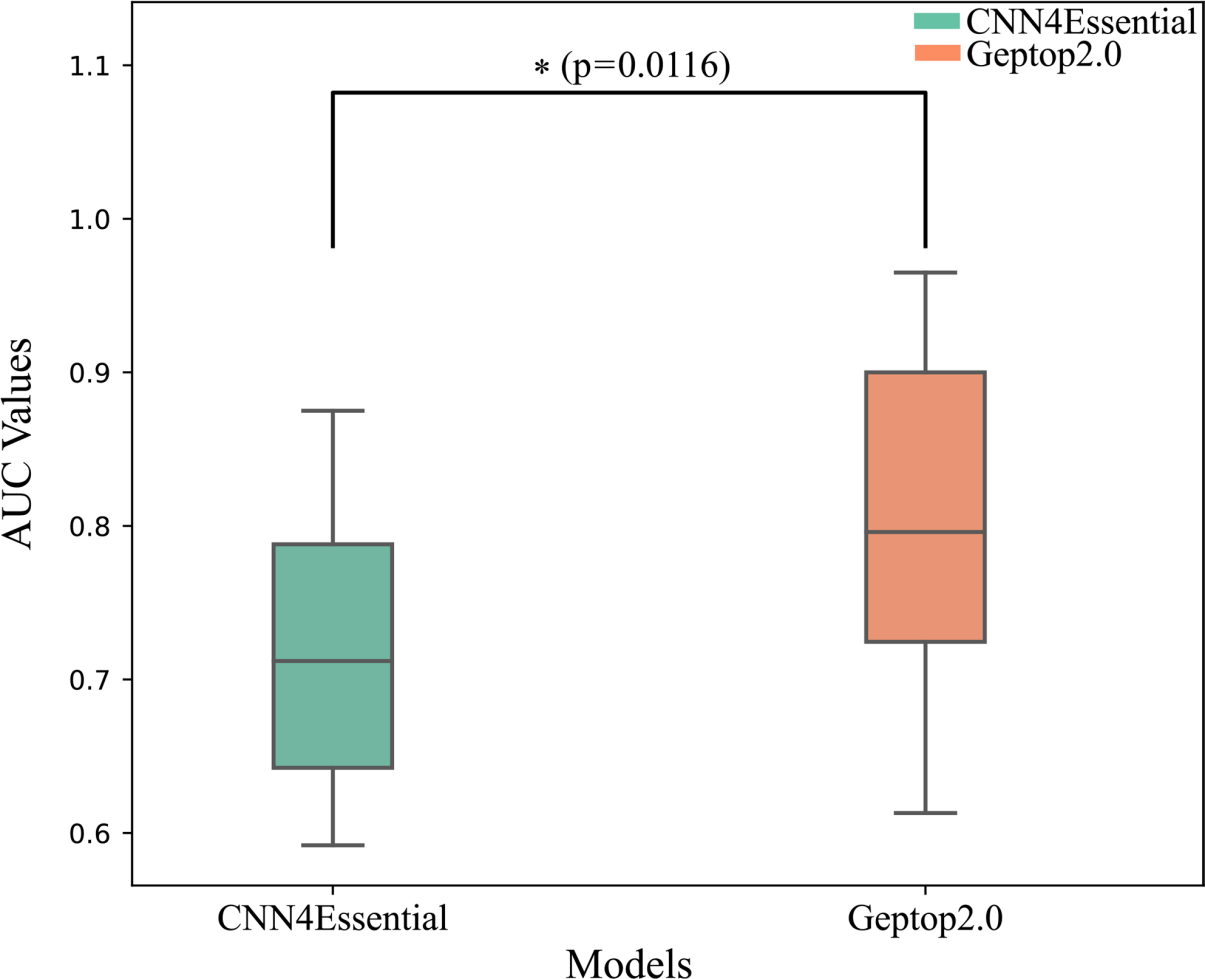
The boxplot comparing the variance of CNN4Essential with Geptop2.0 shows that the asterisk (*) indicates a statistically significant difference between the two results (significance level α = 0.05)

CNN4Essential with 451 features was used for the LASP experiment involving 19 species (species with AUC values > 0.6), achieving an average AUC value of 0.726. We compared the performance of CNN4Essential with the predictive performances of Geptop2.0 [23], information-theoretic features [15], and ANN models [22] reported in the literature. We found that Geptop2.0 achieved a cross-species prediction AUC value of approximately 0.84 across 37 species, whereas the LASP AUC values for information-theoretic features and ANN models are 0.717 and 0.694, respectively, across 37 and 31 species. The AUC value for our LASP model was slightly lower than that of Geptop2.0 (Table 3 and Fig. 3). A variance analysis across 19 species showed CNN4Essential had significantly lower AUC variability than Geptop2.0 (ANOVA, *p* = 0.0116), indicating better performance consistency (Fig. 4).

**Table 3:** Average AUC scores for leave-a-species-out predictions for CNN4Essential model and other models

	AUC	Number of selected features	Number of selected species
CNN4Essential	0.726	451	19
Wen et al. (2019) [23] (Geptop2.0)	0.84	16	37
Liu et al. (2021) [15] (BPNN^a)^)	0.717	114	37
Xu et al. (2020) [22] (ANN^b)^)	0.697	29	31

^a)^*BPNN* Backpropagation Neural Network

^b)^*ANN* Artificial Neural Network

#### CNN4Essential model outperforms other existing methods in all-species prediction

When trained on all species, CNN4Essential achieved strong results: AUC 0.851, accuracy 83.4%, average precision 62.6%, sensitivity 58.8%, specificity 90.6%, and positive predictive value 64.6%. Compared to Nguyen et al.’s [24] ensemble deep neural network (AUC 0.814) and Hasan and Lonardi’s [16] DeeplyEssential model (AUC 0.842), CNN4Essential demonstrated superior performance in Table 4.

**Table 4:** AUC values of self-prediction for all species across different models

	AUC	Accuracy	AP	Sensitivity	Specificity	PPV	Features	Num. of species
CNN4Essential	0.851	0.834	0.626	0.588	0.906	0.646	451	22
Xu et al. (2019) [22] (ANN^a)^)	0.784	-	-	-	-	-	57	31
Md bid Hasan et al. (2019) [16] (DE^b)^)	0.842	0.762	-	0.801	0.721	0.749	89	30
Nguyen Quoc Khanh Le et al. (2020) [24] (EDNN^c)^)	0.814	0.763	-	0.602	0.846	-	100	53

^a)^*ANN* Artificial Neural Network

^b)^*DE* DeeplyEssential

^c)^*EDNN* Ensemble Deep Neural Network, *AP* average precision, *PPV* positive predictive value

### Ablation experiments verify the necessary of feature selection and enhancement

#### Multi-feature fusion to enhance model prediction performance

**Fig. 5:**
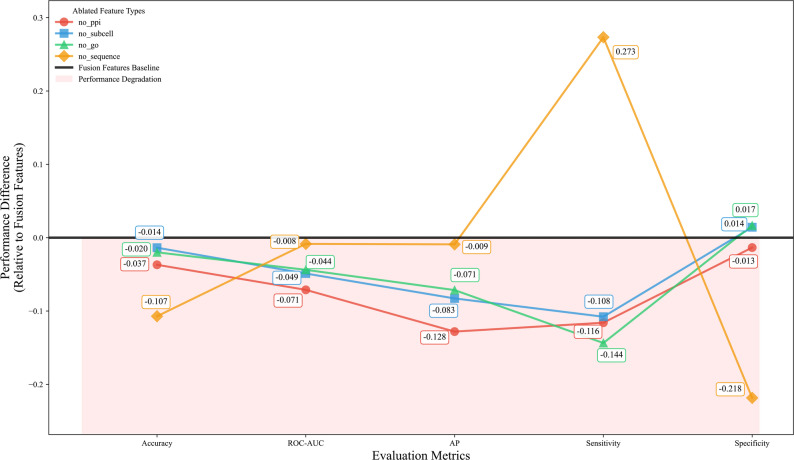
Performance impact of feature ablation on predictive model performance

To explore the roles and contributions of different types of features in the identification task of essential genes, we designed and implemented four feature ablation experiments: removing PPI network features (no_ppi), subcellular localization features (no_subcell), GO annotation information features (no_go), and removing sequence-related features (no_sequence). Figure 5 shows the performance differences in various evaluation metrics of the four feature ablation scenarios compared to the Fusion Features Baseline (all features fused).

The feature ablation experiments demonstrate that the removal of any single feature type (PPI networks, subcellular localization, GO annotations, or sequence features) results in measurable performance degradation across all evaluation metrics, confirming the complementary value of multi-features. The integrated fusion features approach achieved optimal performance, highlighting the importance of combining heterogeneous biological information for accurate predictions.

The elimination of protein-protein interaction (PPI) networks resulted in the most pronounced decrease in ROC-AUC and sensitivity metrics, indicating that inter-protein relational information significantly enhances predictive performance. In a similar manner, the removal of subcellular localization features significantly decreased specificity, underscoring their role in reducing false positive predictions. Gene Ontology annotations consistently demonstrated importance across all metrics. In contrast, sequence features, while still valuable, showed relatively smaller individual contributions, potentially due to partial information redundancy with other features. These findings underscore the significant advantages of employing integrative feature engineering in biological predictive tasks, as it effectively encapsulates various dimensions of biological systems. The enhanced performance observed with fusion features indicates synergistic interactions among different feature types, wherein the collective integration offers greater predictive efficacy than the mere aggregation of individual components. This evidence bolsters the paradigm of multi-feature integration in computational biology, promoting increased model robustness and biological relevance.

### Performance degradation from removing RF weighting and SVD

**Fig. 6:**
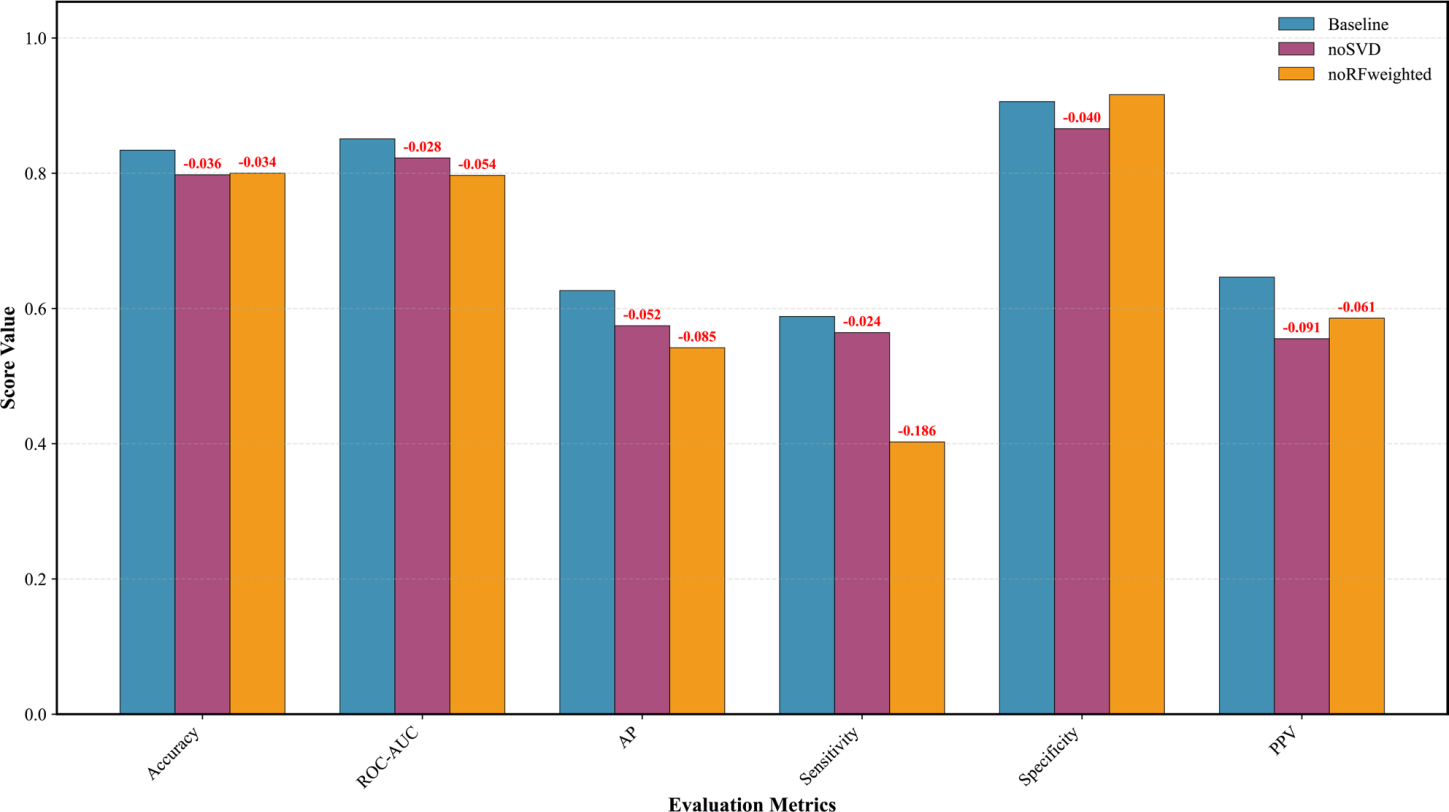
Performance comparison of baseline and ablated models (without RF weighting or SVD). Red labels indicate performance degradation. Both techniques are essential for maintaining model accuracy and robustness

Figure 6 presents a comparative analysis of ablation experiments focused on data processing methodologies. The outcomes of three distinct data processing approaches are integrated into the CNN4Essential architecture for predictive purposes: the baseline approach, which employs both Random Forest (RF) weighting fusion and truncated singular value decomposition; a variant excluding truncated singular value decomposition (noSVD); and another variant excluding RF weighting fusion (noRFweighted). Subsequently, the results are systematically evaluated and compared.

The ablation experiment on data processing procedures reveals that omitting RF weighting and SVD dimensionality reduction leads to substantial declines in performance across various evaluation metrics, thereby affirming their essential roles in the predictive model. Specifically, the exclusion of RF weighting significantly diminishes sensitivity by 18.6% and average precision by 0.085, highlighting its crucial function in addressing class imbalance and improving true positive detection. While the removal of SVD exhibits comparatively milder effects, it consistently results in decreased performance in terms of accuracy, ROC-AUC, and precision, underscoring its importance in reducing feature noise and enhancing model generalization. The findings underscore that RF weighting primarily enhances classification robustness in the context of imbalanced data distributions, whereas Singular Value Decomposition (SVD) contributes to improved feature quality and computational efficiency. The comparative analysis indicates that RF weighting exerts a more pronounced influence on overall model performance, especially concerning recall-related metrics. These results affirm the necessity of integrating both techniques to achieve optimal predictive accuracy and robustness, suggesting that their synergistic application effectively mitigates overfitting and enhances generalization capabilities in complex classification tasks. This study provides empirical evidence that advanced preprocessing and weighting strategies are essential components of contemporary machine learning pipelines, particularly when addressing high-dimensional and imbalanced datasets.

### Comparing the simple baseline models to verify the priority selectivity of CNN4Essential

The predictive performance of the CNN model, which utilized fused features, was evaluated against three baseline models: Random Forest, eXtreme Gradient Boosting, and Logistic Regression. The CNN model demonstrated a distinct selection preference. The comparative results are presented in Table 5.

**Table 5:** Comparison of prediction performance between CNN model and other three simpler baseline models

	CNN4Essential	RF^a)^	XGBoost	LR^b)^
Accuracy	0.834	0.819	0.788	0.584
ROC-AUC	0.851	0.864	0.782	0.659
AP	0.626	0.640	0.498	0.317
Sensitivity	0.588	0.570	0.121	0.747
Specificity	0.906	0.892	0.983	0.537
PPV	0.646	0.607	0.679	0.320
Training time	3–4 h	35–36 h	6–7 h	< 0.5 h

All training time measurements were obtained on the same hardware platform using CPU-only computation: Intel(R) Core(TM) i7-9700 CPU running at 3.00 GHz and 32.0 GB of installed RAM, running a 64-bit Windows operating system

^a)^*RF* Random Forest

^b)^*LR* Logistic Regression, *AP* average precision, *PPV* positive predictive value

### Parameter description of three baseline models

#### Random forest

The classifiers evaluated in this study include Random Forest (RF) models, which were optimized using a grid search approach. These models underwent two iterations of fivefold cross-validation. The optimal hyperparameters identified during the training phase are as follows: 300 estimators, an unrestricted maximum tree depth, the square root of the number of features considered for node splitting, a minimum of two samples required to split an internal node, and a minimum of one sample required to form a leaf node. This configuration yielded the highest Receiver Operating Characteristic - Area Under the Curve (ROC-AUC) score during the cross-validation process.

#### eXtreme gradient boosting

The eXtreme Gradient Boosting (XGBoost) algorithm was employed for the binary classification of gene essentiality. The model was configured with a logistic regression objective function and assessed using logarithmic loss as the evaluation metric. Critical hyperparameters included a maximum tree depth of 6, a learning rate of 0.1, and a feature/row subsampling ratio of 0.8 to mitigate overfitting. The model underwent training via repeated 10-fold cross-validation, repeated five times, with early stopping implemented after 50 consecutive rounds without improvement. The optimal classification threshold was identified by maximizing Youden’s J statistic across the validation folds, with the median threshold ultimately selected for application.

#### Logistic regression

The logistic regression model (LR) is employed to predict gene necessity based on an array of comprehensive genomic features. The model is trained using a balanced methodology and utilizes k-fold cross-validation to determine a robust classification threshold. Feature normalization is implemented to ensure consistent scaling across all input variables. The final model enhances the performance for both essential and non-essential gene categories by incorporating L2 regularization and balanced class weights. The optimal classification threshold is identified during cross-validation by maximizing Youden’s J statistic at 0.5, thereby ensuring balanced sensitivity and specificity.

The CNN4Essential model is preferred for predicting bacterial gene essentiality due to its excellent balance of performance and computational efficiency. While the RF classifier slightly outperforms it in ROC-AUC (0.864 vs. 0.851) and Average Precision (0.640 vs. 0.626), CNN4Essential is more practical. It offers significantly faster training and greater computational efficiency, unlike the RF method, which requires extensive resources to build and combine numerous decision trees, particularly with large genomic datasets. CNN4Essential achieved the highest Accuracy (0.834) and Positive Predictive Value (0.646), demonstrating strong predictive power and reliability in identifying essential genes. It balances Sensitivity (0.588) and Specificity (0.906), minimizing error bias. Its scalability and efficiency make it ideal for high-throughput genomic applications requiring speed and accuracy. Thus, despite slightly lower scores in two metrics, CNN4Essential is a more sustainable and practical choice for genome-wide essentiality prediction.

### Case study: *Haemophilus influenzae* and antimicrobial target identification

*Haemophilus influenzae* is a Gram-negative bacterium commonly found in the human upper respiratory tract [25]. It is capable of causing various clinical infections, including otitis media, pneumonia, meningitis, laryngitis, and sepsis. By identifying potential drug target genes, more precise and effective strategies can be developed for treating *H. influenzae* infections, particularly in combating drug resistance and chronic infections. Therefore, this study focuses on *H. influenzae* as an example, following the materials and methods described earlier to collect, preprocess, extract features, fuse features, and perform dimensionality reduction on the species’ data. The proposed CNN4Essential universal recognition model is then used to predict essential genes of *H. influenzae*. The predicted essential genes are matched with drug target genes for the bacterium collected from the DrugBank database [26] to explore the potential of predicting essential bacterial genes as antimicrobial drug targets.

**Fig. 7:**
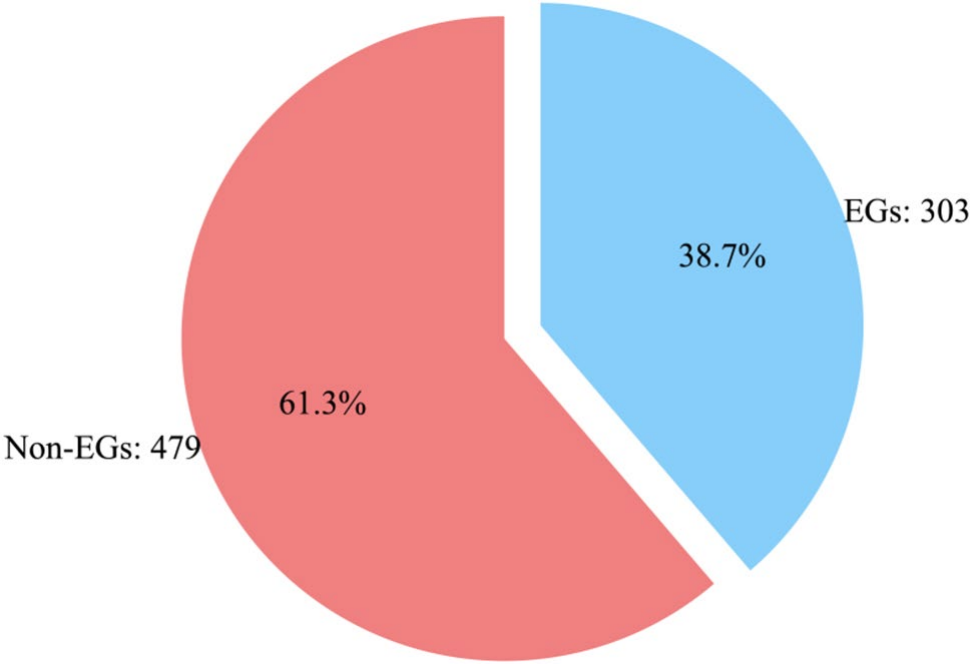
Number and proportion of positive and negative samples collected from *H. influenza*

Following the Materials and Methods section, gene data for *H. influenzae* was downloaded, and the necessary feature data for the model was collected. Figure 7 displays the count of positive and negative samples. A weighted fusion model optimizes feature weights and matrix decomposition for dimensionality reduction, enhancing the quality of feature data. The pre-trained CNN4Essential universal model is called, and the processed feature data, along with classification labels, are input into the model for prediction. The prediction results are then evaluated.

**Fig. 8:**
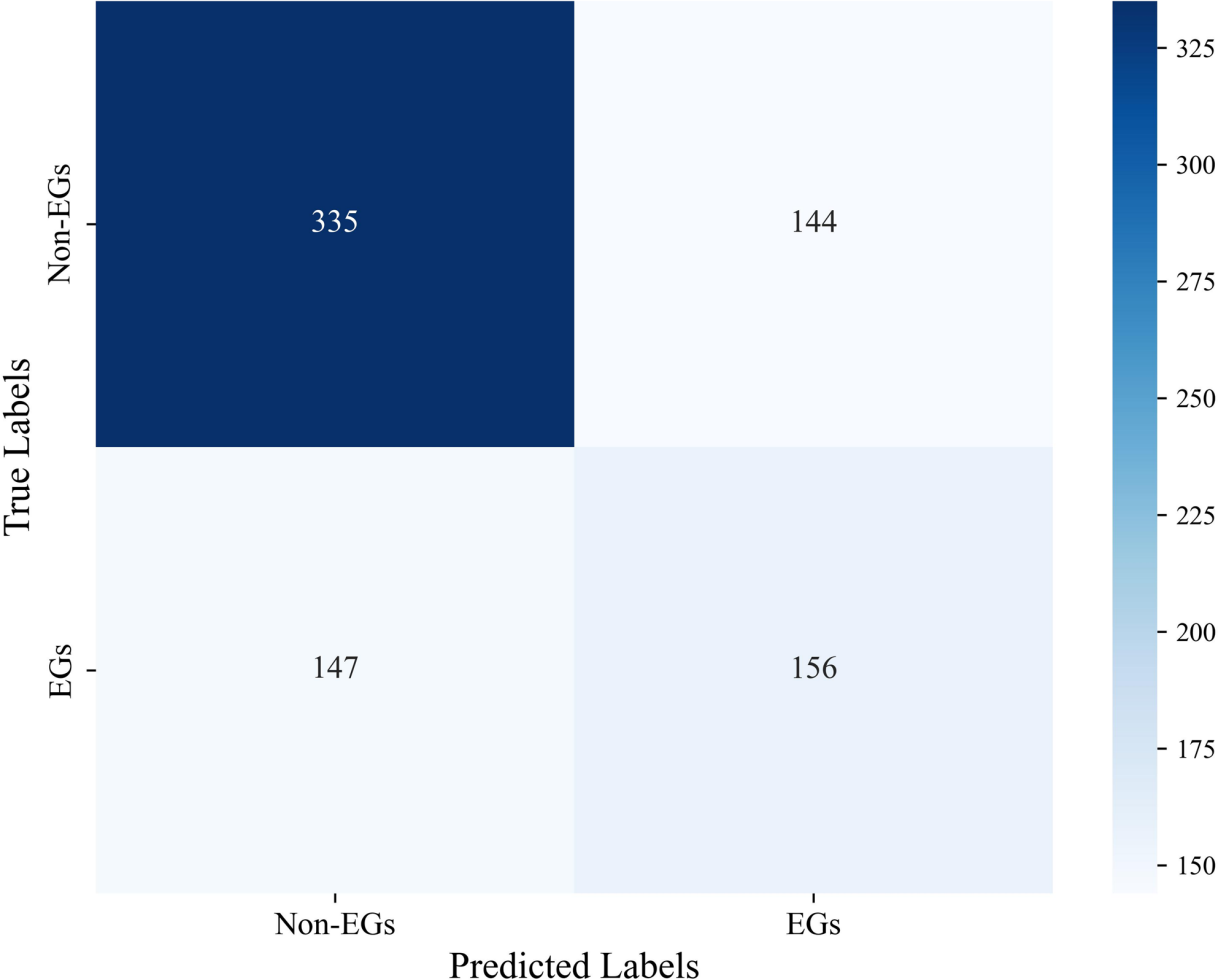
Confusion matrix for predicting and evaluating essential genes in *H. influenza*

The reliable dataset obtained from dimensionality reduction is used as the test set for predicting the gene essentiality of *H. influenzae* with the trained CNN4Essential model, resulting in the confusion matrix shown in Fig. 8. From the figure, it is clear that most Non-Essential Genes (Non-EGs) were correctly identified, with more than half of the Essential Genes (EGs) correctly identified. There are 303 EGs in the original data, and the predicted EGs are close to 303, reflecting the effectiveness of the prediction. The main evaluation metric, AUC, is 0.606, which is 3.8% higher than the performance of LASP.

**Fig. 9:**
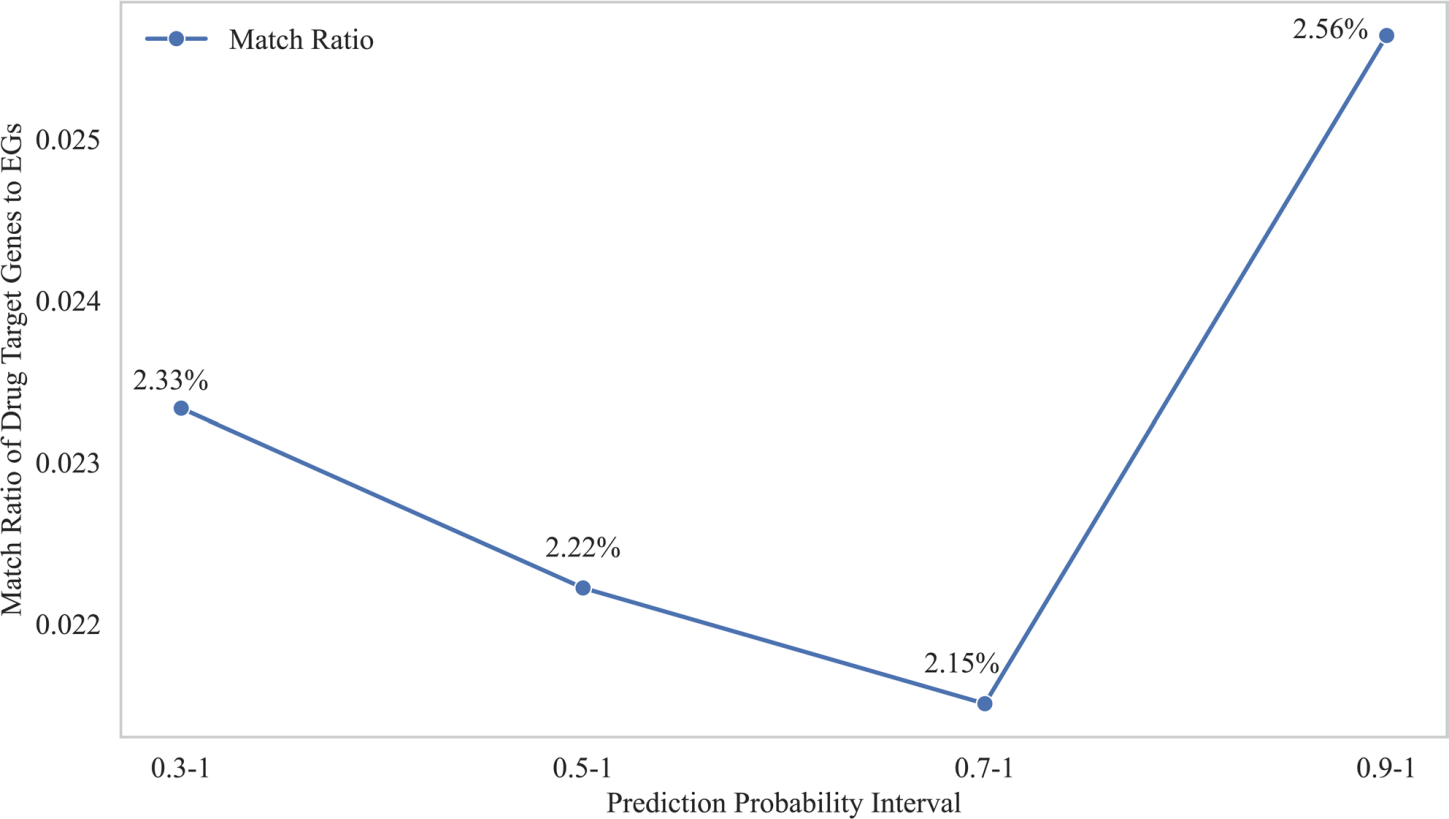
Tendency of the Matching Ratio for Potential Antimicrobial Target Genes across Model Confidence Thresholds

Due to the imbalance of positive and negative samples in the *H. influenzae* dataset, a threshold of 0.3 was used for classification, and the scores of predicted Essential Genes (EGs) were grouped into ranges: 0.3–0.5, 0.5–0.7, 0.7–0.9, and 0.9-1. The essential gene with the highest predicted score is *gyrA*, with a score of 0.955; the number of identified essential genes that overlap with drug target genes verified in DrugBank is shown in Table 6. The model prioritization capability was assessed. We analyzed the Matching Ratio for Potential Antimicrobial Target Genes among predicted essential genes. This analysis was done across increasing confidence thresholds. Figure 9 depicts the relationship between the model’s prediction probability interval (x - axis) and this matching ratio (y - axis). This matching ratio is defined as the proportion, within each probability interval, of predicted essential genes that are confirmed antimicrobial targets in DrugBank.

**Table 6:** The gene names and functional annotations that are identified as essential genes in each scoring segment coincide with those confirmed as drug target genes in DrugBank

Score_Range	Gene_Name	functional_annotations	Predicted_Probability
0.3–0.5	aroE	NADP binding	0.382
	cysE	serine O-acetyltransferase activity	0.317
	trmD	tRNA (guanine(37)-N1)-methyltransferase activity	0.387
0.5–0.7	rpiA	ribose-5-phosphate isomerase activity	0.622
	rplD	rRNA binding	0.5145
0.7–0.9	yciA	long-chain fatty acyl-CoA hydrolase activity	0.725
0.9-1	gyrA	ATP binding	0.995

As shown in Fig. 9, the Matching Ratio remains at a low and stable level within the broad to mid - range probability thresholds (0.3, 0.5, and 0.7). This indicates that although the model creates a large candidate pool, the proportion of genes matching established drug targets is limited. This is consistent with the biological complexity related to gene essentiality. Many genes may be predicted by computation as essential under specific conditions but have not been used as drug targets because of various pharmacological limitations. The key finding is the obvious upward shift in the Matching Ratio within the highest confidence interval (0.9–1.0). The curve shows a clear inflection point, at which the ratio reaches its maximum value. This definitive upward trend signifies that the subset of genes assigned the highest confidence scores by our model is enriched for genuine, pharmaceutically relevant antimicrobial targets.

The observed trend provides a strong confirmation of our model’s utility in candidate prioritization. At stringent thresholds, the rising Matching Ratio suggests that the model successfully identifies genes that are both essential and druggable. These high - probability genes likely encode key non - overlapping components of critical bacterial survival pathways, precisely the type of targets that are most susceptible to chemical inhibition and have had a high success rate in the history of antibiotic development.

From a translational perspective, this increasing Matching Ratio curve provides a powerful strategy for allocating experimental resources. By focusing on top - tier predictions (e.g., prediction probability ≥ 0.9), researchers can define a small, high - priority candidate set more likely to contain viable targets. This approach efficiently filters out a large number of lower - probability candidates, thus optimizing the cost and time efficiency of downstream target validation studies.

In summary, the trajectory of the Matching Ratio clearly shows that the model’s primary strength is its ability to rank and prioritize candidates based on their likelihood of being successful antimicrobial targets. The significant uplift in the ratio at the highest confidence interval provides a compelling data - driven rationale for setting a high probability threshold to identify promising candidates for subsequent experimental investigation, which ultimately accelerates the early stages of antibiotic discovery.

## Discussion

The differences in INSP performance across species can be attributed to varying data quality and genome complexity. For example, STY had more training genes and distinct genomic characteristics, making learning more effective. Conversely, SEL had less genes and a more complex genome, with less distinguishable features. To further support this observation, we examined the relationship between the training sample size and INSP performance across all 22 species. A significant positive correlation was observed between the total number of genes used for training each species and the corresponding AUC values (Spearman’s ρ = 0.565, *p* = 0.0062; Supplementary Figure S1), indicating that species with fewer available training samples generally exhibit lower predictive performance. Additionally, biological characteristics such as gene expression, metabolism, and environmental adaptation may also influence model performance.

In LASP, the decline in predictive accuracy likely stems from increased heterogeneity in the combined training data. Incorporating diverse species may introduce noise, affect feature representativeness, and reduce generalizability—especially when test species are evolutionarily distant from those in training. CNN4Essential’s relatively strong LASP performance reflects its robustness to such heterogeneity, likely due to the integration of diverse biological features.

Compared to shallow models and traditional approaches, CNN4Essential benefits from feature diversity (sequence, interaction, annotation), high-dimensional architecture, and advanced preprocessing techniques like normalization and SMOTE. These factors collectively enhance performance, particularly for species lacking strong evolutionary ties to others.

**Fig. 10:**
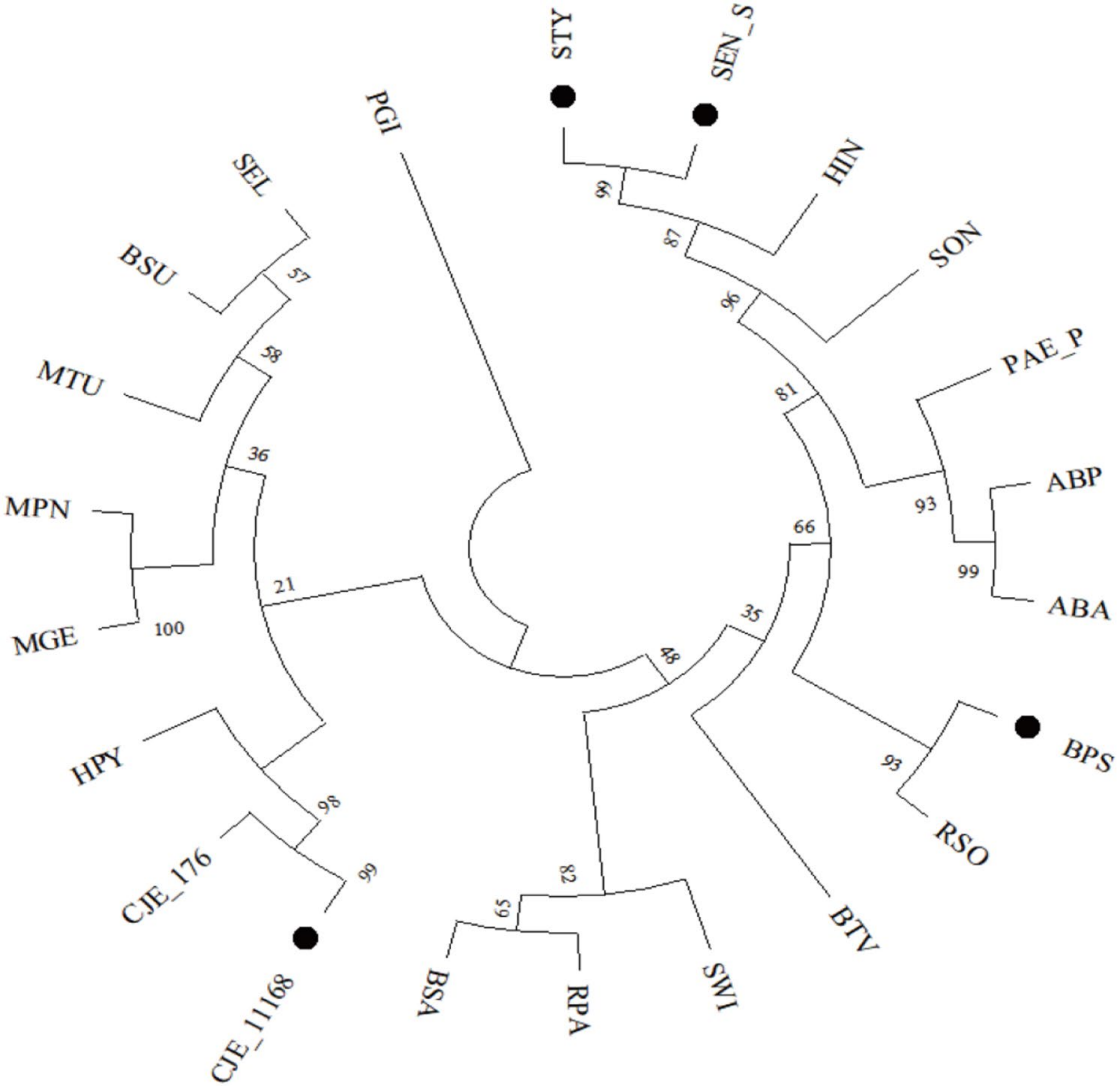
Phylogenetic tree of 22 bacterial species used for essential gene prediction analysis. The phylogenetic tree illustrates the evolutionary relationships among the 22 bacterial species analyzed. The tree was constructed based on genomic data, with bootstrap values displayed at major nodes to indicate the confidence of branching. Species marked with black dots (e.g., STY, BPS, CJE_11168, SEN_S) represent the four species for which the proposed model achieved higher AUC values compared to Geptop2.0. These species are positioned in separate branches, highlighting their evolutionary divergence. The remaining 18 species are distributed across various branches, often distant from the marked species, reflecting substantial genetic diversity

Although Geptop2.0 showed slightly higher LASP AUC, its higher variance and long runtime (> 30 min/species) limit practical applications. In contrast, CNN4Essential is more efficient and consistent. Moreover, Geptop2.0 relies heavily on evolutionary data, while CNN4Essential remains effective for phylogenetically distant species (Fig. 10), making it better suited for novel bacterial gene prediction tasks.

Finally, the case study on H. influenzae illustrates the model’s potential in drug target discovery. As prediction probabilities increased, the likelihood of overlap with known drug targets improved, especially in the 0.9–1 range. This suggests that CNN4Essential can prioritize high-confidence targets, offering an efficient strategy to support antimicrobial development.

## Conclusions

In this study, we systematically evaluated the predictive capabilities of essential genes across different bacterial species using a convolutional neural network (CNN) model based on multi-feature information fusion, with a particular focus on the intra-species prediction (INSP) and leave-a-species-out prediction (LASP) performances. In the INSP experiments, the model achieved an average AUC of 0.884, indicating excellent predictive performance, The differences in predictive accuracy across species were significantly influenced by quality and quantity of data.

We found that the performance differences of the CNN model were not only related to sample size but were also closely linked to the genomic complexity and biological characteristics of the bacteria. The STY genome is relatively simple and distinct, which allowed the model to effectively capture key features. Conversely, the SEL genome is more complex, containing substantial redundant information, which presented greater challenges for the model during the learning process. Biological differences among bacteria, such as gene expression patterns, metabolic pathways, and environmental adaptability, also may contribute to variations in the CNN model’s ability to identify essential genes.

In the LASP experiments, although the CNN model achieved an average AUC of 0.726, which is lower than that of the Geptop2.0 model (AUC approximately 0.84), our CNN model demonstrated good predictive potential by integrating multiple features. The results of the LASP experiments highlighted the influence of factors such as evolutionary distance between species, feature consistency, and training data diversity on predictive performance. Particularly, when the training set included data from multiple species, the CNN model faced increased risks of noise and insufficient feature representativeness, which may lead to biased predictions.

We compared the predictive performance of our CNN model with that of related models, and demonstrated the advantages of our CNN model in feature set diversity and deep learning model architecture. Compared with existing studies that rely on a single feature type or shallower models, our CNN model exhibited significant capabilities in recognizing and processing multidimensional features. The findings also indicate that data preprocessing steps, such as normalization and sample balancing, may help to improve predictive accuracy. Notably, in the predictions across all species, our CNN model achieved an AUC of 0.851 and accuracy of 83.4%, which not only demonstrates the advantages of deep learning methods in gene prediction tasks but also emphasizes the importance of multi-feature biological information fusion. By integrating information from diverse biological sources, the CNN model comprehensively captures gene features, which enhances the robustness and accuracy of the predictions.

In summary, the CNN model developed in this study demonstrated exceptional performance in predicting essential genes in bacteria and laid a solid foundation for future research. Future work will focus on optimizing feature selection strategies and integrating additional biological information to enhance the model’s generalization ability and practical applicability. We anticipate that these improvements will provide robust tools for research on bacterial genes, thereby contributing to advancements in biomedicine and microbiology.

## Materials and methods

### Experimental design

**Fig. 11:**
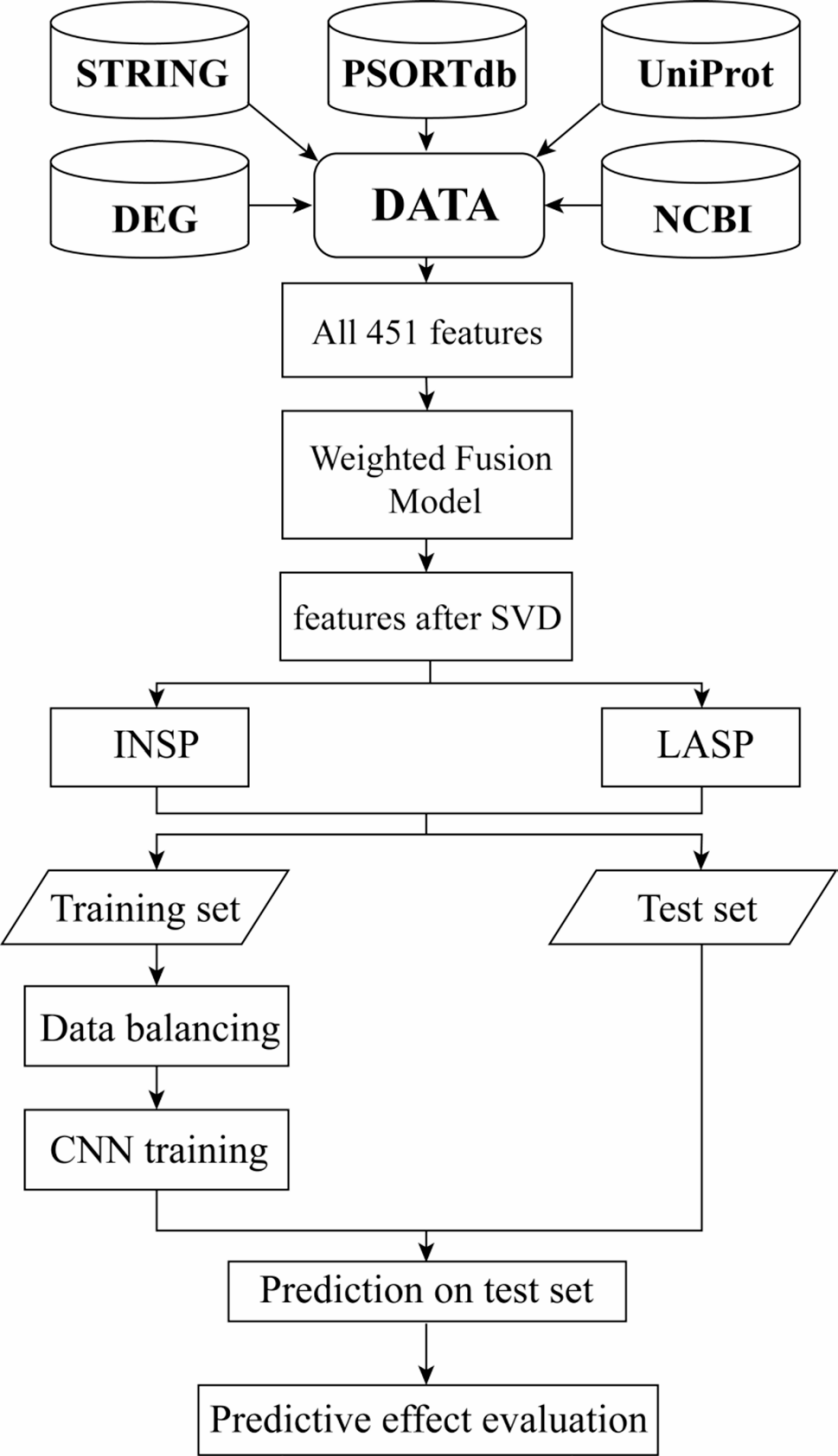
Technical workflow and overview of the study design. Two approaches, intra-species prediction (INSP) and leave-a-species-out prediction (LASP), are used to evaluate the proposed method. DEG, Database of Essential Genes; NCBI, National Center for Biotechnology Information database; STRING, Search Tool for the Retrieval of Interacting Genes/Proteins; UniProt, Universal Protein Resource database; SVD, singular value decomposition; CNN, convolutional neural network

Essential gene prediction models were constructed based on the selected features and used to predict essential genes in 22 prokaryotic species. The technical workflow and overview of the study design are illustrated in Fig. 11.

### Datasets

Essential gene sequences and the amino acid sequences of bacterial species were downloaded from the Database of Essential Genes (DEG) [27]. The RefSeq accession numbers (e.g., NC_000964) provided in DEG were used to retrieve all the genes of the bacterial species from the National Center for Biotechnology Information (NCBI) database [28]. The gene sequences and corresponding amino acid sequences were downloaded from the NCBI databases. The genes were classified based on their essentiality in the bacterial species.

**Fig. 12:**
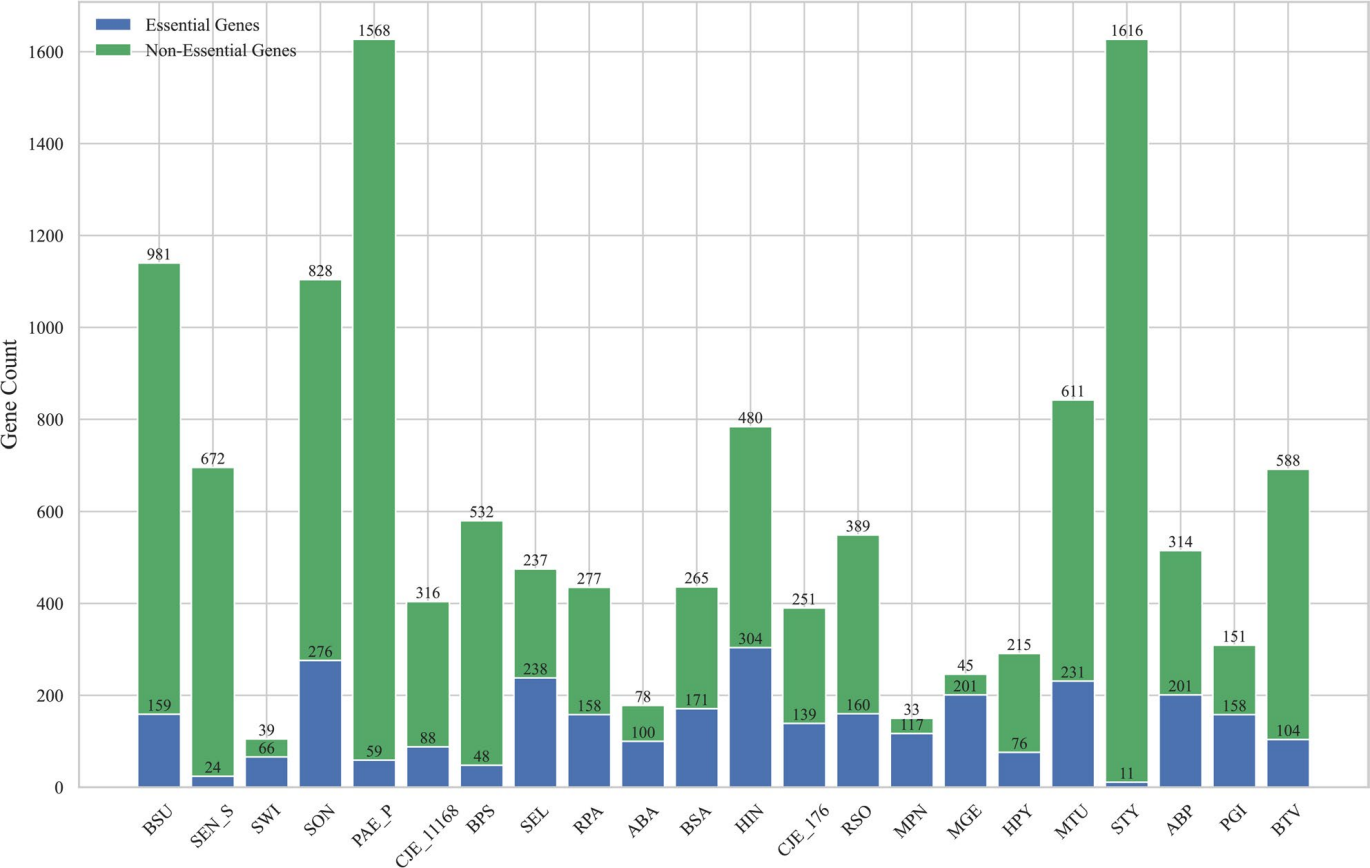
The number of EGs and Non-EGs in 22 bacterial species

Information on bacterial protein–protein interaction (PPI) networks was obtained by searching and downloading from STRING, the Search Tool for the Retrieval of Interacting Genes/Proteins [29], using the species name. Gene annotation information under the three Gene Ontology (GO) categories—biological process, cellular component, and molecular function—was retrieved and downloaded from the Universal Protein Resource (UniProt) database [30] using the species name. The bacterial subcellular localization data used in this study were obtained using the computational prediction module cPSORTdb in the PSORTdb database [31]. This is because experimentally validated bacterial subcellular localization information was available for only a limited number of species. The number of essential genes (EGs) and non-essential genes (Non-EGs) collected for the 22 species is shown in Fig. 12.

### Feature calculation

#### Sequence and sequence-derived features

Sequence features, namely sequence length, GC content, molecular weight, and protein instability index, were calculated for the collected gene sand amino acid sequences. Primary sequence features, namely mononucleotide frequency (monomer, 4 variables), phased dinucleotide frequency (dimer, 16 variables), phased trinucleotide frequency (trimer, 64 variables), and phased tetranucleotide frequency (tetramer, 256 variables), were determined. We also extracted the minimum free energy (MFE) of each gene’s coding sequence as an input feature. This feature stems from a biological hypothesis: mRNA transcripts of essential genes may evolve to have more stable secondary structures (indicated by lower MFE), and thus, the calculated MFE value becomes a selected sequence feature. The MFE of each mRNA transcript was computed using the ViennaRNA Package (version 2.5.1) [32] under standard thermodynamic conditions. Calculations were performed using the RNA.fold_compound() and .mfe() methods with the default parameters, which implement the Turner 2004 energy model [33] at 37 °C, with dangling end contributions on both ends of a helix (dangles = 2), special hairpin loops enabled, and the possibility of lonely base pairs (noLP = 0). Finally, we obtained 345 sequence and sequence-derived features for further analyses.

### Interaction network features of gene-encoded proteins

A PPI network was constructed using the downloaded protein interaction information, and key network parameters such as node degree and clustering coefficient were calculated. To understand the connectivity preferences of gene-encoded proteins—specifically their connections to essential or non-essential genes—total number of interacting proteins, number of essential gene-encoded proteins, and number of non-essential gene-encoded proteins were quantified. Overall, five features were derived from the PPI network.

### Gene annotation information features

Annotation information from the three GO categories—biological process, cellular component, and molecular function—was transformed into 100-dimensional vectors using the Word2Vec method [34]. A total of 100 features were obtained from this analysis.

### Subcellular localization features

The subcellular localization information of gene-encoded proteins was obtained for each bacterial species. Different localization positions were mapped to numerical values using integer encoding. Ultimately, one feature for subcellular localization was obtained.

### Feature fusion

#### Weighted fusion model

To effectively integrate multiple sets of biological features and improve classification performance, we used a weighted fusion model based on feature importance calculated by a random forest (RF) algorithm. By assigning different weights to different features, effective fusion of multi-dimensional features was achieved. First, all the features are standardized to eliminate discrepancies in scales. Then, the importance of each feature type is calculated using the RF model; weights are determined based on the mean importance values. Finally, the features are fused using a weighted approach to ensure that the contributions of different features are reasonably reflected in the classification task.

Feature standardization is calculated as:

1 $${X_{{\mathrm{scaled}}}}=\frac{{X - \mu }}{\sigma }$$

where *X* is the original feature, *µ* is the feature’s mean, and *σ* is the standard deviation. This step ensures that the mean of each feature is zero and the variance is one.

Subsequently, the importance of each feature type is calculated using a RF classifier. RF measures feature importance based on the reduction in the Gini coefficient, and is calculated as:

2 $${\mathrm{Importance}}({f_i})=\frac{1}{T}\sum\limits_{{t=1}}^{T} {\Delta Gini({f_i},t)} $$

where *T* is the number of trees in the RF and $\Delta Gini({f_i},t)$ is the reduction in the Gini coefficient caused by feature *f*_*i*_ in decision tree *t*. The importance of each feature type is calculated using this method, and the mean importance value for each feature type is obtained by averaging

3 $$\overline {{{\mathrm{Importance}}}} {\mathrm{=}}\frac{1}{n}\sum\limits_{{i=1}}^{n} {{\mathrm{Importance}}({f_i})} $$

where 𝑛 is the total number of features in that category.

Next, the feature weights are calculated based on the mean importance of each feature type. The weight ${w_{{\mathrm{category}}}}$ represents the relative contribution of each feature type to the overall model, and is calculated as:

4 $${w_{{\mathrm{category}}}}=\frac{{{{\overline {{{\mathrm{Importance}}}} }_{{\mathrm{category}}}}}}{{\sum\nolimits_{{{\text{all categories}}}} {\overline {{{\mathrm{Importance}}}} } }}$$

Finally, the features are fused based on their importance weights. The fused feature matrix ${X_{{\mathrm{fused}}}}$ is calculated as:

5 $${X_{{\mathrm{fused}}}}={w_{{\mathrm{sequence}}}}{X_{{\mathrm{sequence}}}}+{w_{{\mathrm{ppi}}}}{X_{{\mathrm{ppi}}}}+{w_{{\mathrm{subcell}}}}{X_{{\mathrm{subcell}}}}+{w_{{\mathrm{go}}}}{X_{{\mathrm{go}}}}$$

### Feature matrix dimension reduction

In high-dimensional data analysis, feature dimensionality reduction is an important preprocessing step aimed at preserving the main data information by reducing the number of features, thereby improving the efficiency and accuracy of subsequent analyses. Particularly in machine learning and pattern recognition, dimensionality reduction not only reduces the computational complexity but also decreases the risk of overfitting. In this study, we used the truncated singular value decomposition method for feature dimensionality reduction.

Singular value decomposition is a powerful matrix factorization technique that decomposes any arbitrary *m*×*n* matrix *X* into the product of three matrices:

6 $$X=U\Sigma {V^T}$$

where *U* is an *m*×*m* orthogonal matrix known as the left singular matrix, $\Sigma$ is an *m*×*n* diagonal matrix with singular values as its diagonal elements, and *V* is an *n*×*n* orthogonal matrix known as the right singular matrix.

In the dimensionality reduction process, the aim is not to retain all singular values but rather to keep only the largest *k* singular values, resulting in a truncated singular value decomposition:

7 $$X \approx {U_k}{\Sigma _k}{V_k}^{T}$$

where ${U_k}$ is an *m*×𝑘 matrix composed of the first 𝑘 left singular vectors, ${\Sigma _k}$ is a 𝑘×𝑘 diagonal matrix containing only the largest 𝑘 singular values, and ${V_k}$ is an *n*×𝑘 matrix composed of the first *k* right singular vectors. To quantify the amount of information retained, we define the explained variance ratio (EVR) as:

8 $$EVR=\frac{{\sum\nolimits_{{i=1}}^{k} {{\sigma _i}^{2}} }}{{\sum\nolimits_{{i=1}}^{r} {{\sigma _i}^{2}} }}$$

where ${\sigma _i}$ is the *i*-th singular value, *k* is the number of singular values chosen to be retained, and *r* is the rank of the original data matrix. By iterating over different values of *k*, we calculate the cumulative EVR corresponding to each *k* as:

9 $$EVR{\text{ for }}k=\sum\limits_{{i=1}}^{k} {\frac{{{\sigma _i}^{2}}}{{\sum\nolimits_{{j=1}}^{r} {{\sigma _j}^{2}} }}} $$

The value of *k*, was determined based on maximally retaining the original features. Using this method, truncated singular value decomposition not only effectively reduces data dimensionality but also retains the most important information components from the original data, thereby providing a solid foundation for subsequent training of predictive models. We used the synthetic minority oversampling technique to balance positive and negative samples in the training data, which enhances diversity by generating new samples for the minority class.

### Prediction model establishment

**Fig. 13:**
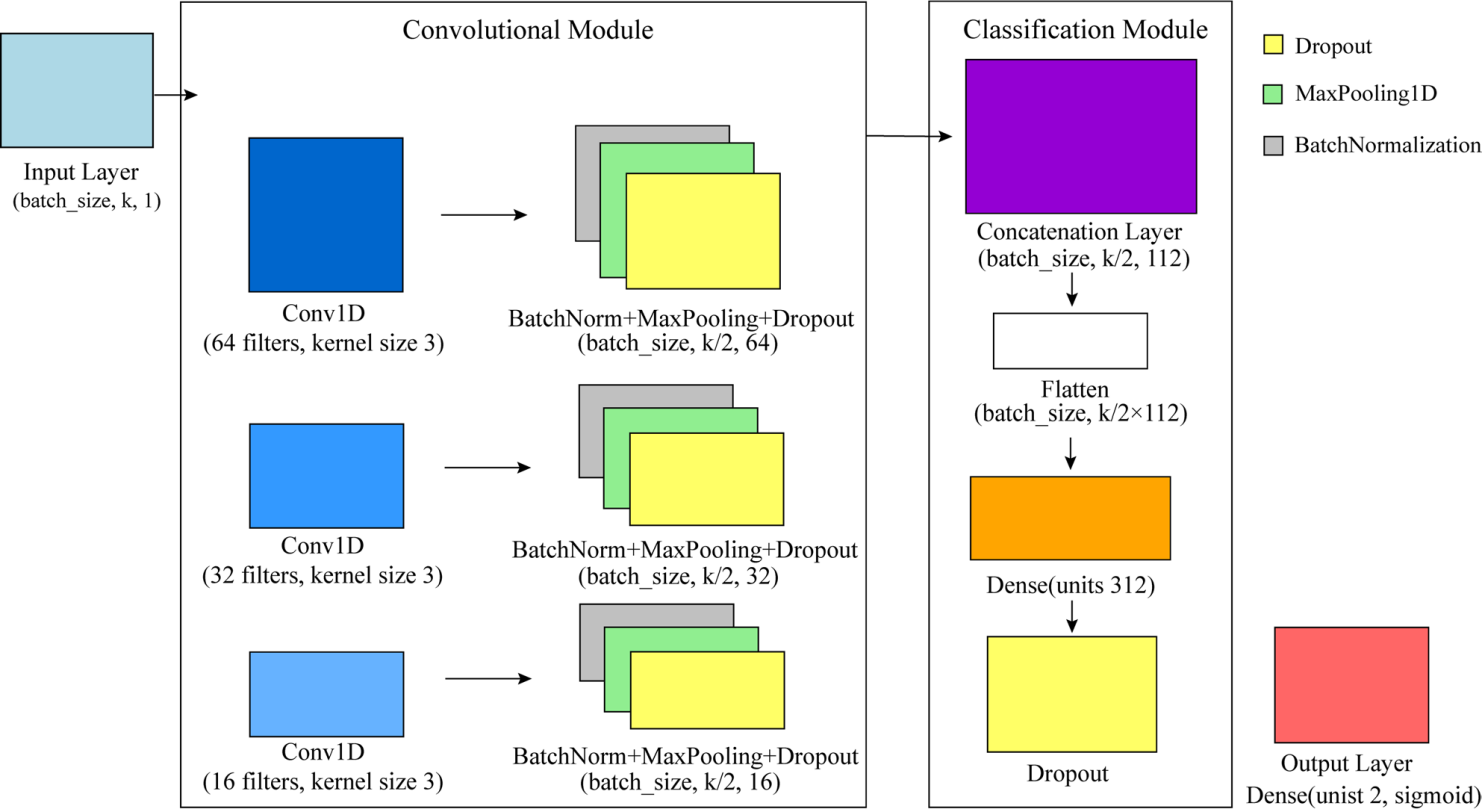
Architecture of the proposed convolutional neural network model. *k* is the number of retained features after RF weighting and SVD

Although convolutional neural networks (CNNs) were originally designed for image processing, they have demonstrated significant potential in handling structured data such as time series, tabular data, and sensor data. CNNs are particularly effective in the automatic extraction of features. In this study, we constructed a CNN model comprising an input layer, three convolutional layers, and an output layer as illustrated in Fig. 13. By incorporating four types of feature data as input to the model, the entire prediction model is named CNN4Essential. Perform full-balance Synthetic Minority Over-Sampling Technique (SMOTE) data sampling on the divided training set before model training to ensure that the model fully learns feature information. SMOTE was applied only to the training data within each fold of the cross-validation process, while the validation/test data remained unchanged to avoid any potential data leakage. During model training, the Adam optimizer was used with a fixed learning rate of 0.001. The training used a batch size of 32. It was carried out for a maximum of 100 epochs. An early - stopping mechanism with a patience of 10 epochs was incorporated to mitigate overfitting. To further enhance generalization, L2 regularization with a coefficient of 0.01 was applied to both convolutional and dense layers. Dropout was applied at rates of 0.25 after convolutional layers and 0.5 in the fully connected layer.

The input data format for the CNN model consists of structured data, including ${n_{{\mathrm{features}}}}$ features. The input tensor is denoted as $X \in {R^{{\text{batch size}} \times {n_{{\mathrm{features}}}}}}$, representing a batch of samples with batch_size, where each sample contains ${n_{{\mathrm{features}}}}$ features. The convolution operation in the convolutional layer captures local dependencies between features. One-dimensional convolution extracts neighboring features within a fixed window. The convolutional kernel is calculated based on the number of features and the size of the convolutional kernel as:

10 $${y^{(k)}}(t)=\sum\limits_{{i=0}}^{2} {{w_i}^{{(k)}}x(t+i)+{b^{(k)}}} $$

where, *x*(*t*) is the *t*-th feature value and *w*_*i*_^(*k*)^ is the weight of the *k*-th convolutional kernel. Zero-padding is applied after each convolution operation to ensure that edge information is preserved. The pooling layer is used for downsampling along the feature dimension, selecting the maximum value within a local window to capture the most important features, thereby reducing computational complexity. When the pooling window size is 2, the feature dimension is halved. The pooling operation is expressed as:

11 $$y(t)=\hbox{max} (x(2t),x(2t+1))$$

The batch normalization maintains stable activation values at each layer during training, thereby accelerating convergence. This process is expressed as:

12 $${\hat {x}^{(i)}}=\frac{{{x^{(i)}} - {\mu _B}}}{{\sqrt {{\sigma ^2}_{B}+\varepsilon } }}$$

where, ${\mu _B}$ and ${\sigma ^2}_{B}$ are the mean and variance of the batch, respectively, and $\varepsilon$ is a very small constant.

To prevent overfitting during training, dropout is applied to the output of each layer to enhance the model’s generalization ability. After a series of convolution, pooling, and batch normalization operations, the resulting output is merged and passed to the fully connected layer:

13 $$y=\sigma (Wx+b)$$

where, *W* is the weight matrix, *b* is the bias term, *x* is the output from the previous layer, and *σ* is the rectified linear unit (ReLU) activation function.

Finally, the output layer uses two neurons, and the output is compressed into the [0,1] range using the sigmoid activation function. The output is a two-dimensional vector that represents the probabilities of the classification into essential and non-essential genes as:

14 $$sigmoid(x)=\frac{1}{{1+{e^{ - x}}}}$$

### Evaluation metrics

The performance evaluation metrics for essential gene identification models primarily include the area under the ROC curve (AUC) value. Additional evaluation metrics are accuracy, average precision (AP), sensitivity, specificity, and positive predictive value (PPV).

15 $$AUC=\int_{0}^{1} {TPR(FPR)d(FPR)} $$

16 $$Accuracy=\frac{{TP+TN}}{{TP+TN+FP+FN}}$$

17 $$AP=\sum\limits_{n} {({R_n} - {R_{n - 1}}){P_n}} $$

  

18 $$Sensitivity=\frac{{TP}}{{TP+FN}}$$

19 $$Specificity=\frac{{TN}}{{TN+FP}}$$

20 $$PPV=\frac{{TP}}{{TP+FP}}$$

These six evaluation metrics are commonly used in binary classification tasks to assess the performance of models.

## Supplementary Information

Supplementary Material 1.

Supplementary Material 2.

Supplementary Material 3.

## Data Availability

Data are openly available in a public repository. The data that support the findings of this study are openly available in DEG [http://origin.tubic.org/deg/public/index.php], NCBI [https://www.ncbi.nlm.nih.gov/], STRING [https://string-db.org/], UniProt [https://www.uniprot.org/], and PSORTdb [https://db.psort.org/].

## Abbreviations

ACC: Accuracy
ANN: Artificial Neural Network
ANOVA: Analysis of Variance
AP: Average Precision
AUC: Area Under the Receiver Operating Characteristic Curve
BPNN: Backpropagation Neural Network
CNN: Convolutional Neural Network
CNN4Essential: Convolutional Neural Network for Essential Gene Prediction
DE: DeeplyEssential
DEG: Database of Essential Genes
EDNN: Ensemble Deep Neural Network
EGs: Essential Genes
EVR: Explained Variance Ratio
GO: Gene Ontology
INSP: Intra-species Prediction
LASP: Leave-a-Species-Out Prediction
LR: Logistic Regression
MFE: Minimum Free Energy
NCBI: National Center for Biotechnology Information
Non-EGs: Non-essential Genes
PPI: Protein–Protein Interaction
PPV: Positive Predictive Value
PSORTdb: Protein Subcellular Localization Database
RefSeq: Reference Sequence Database
RF: Random Forest
ROC: Receiver Operating Characteristic
SMOTE: Synthetic Minority Over-sampling Technique
STRING: Search Tool for the Retrieval of Interacting Genes/Proteins
SVD: Singular Value Decomposition
UniProt: Universal Protein Resource
XGBoost: eXtreme Gradient Boosting
