# Supplementary material for: CNN4Essential: a convolutional neural network model for predicting bacterial gene essentiality based on multi-feature fusion
Source: BMC Genomics. 2026 Apr 6;27:496. doi: 10.1186/s12864-026-12819-3 (PMC13188266; doi:10.1186/s12864-026-12819-3)
Supplement: Supplementary file 2 — Supplementary Material 2. [file 12864_2026_12819_MOESM2_ESM.docx]

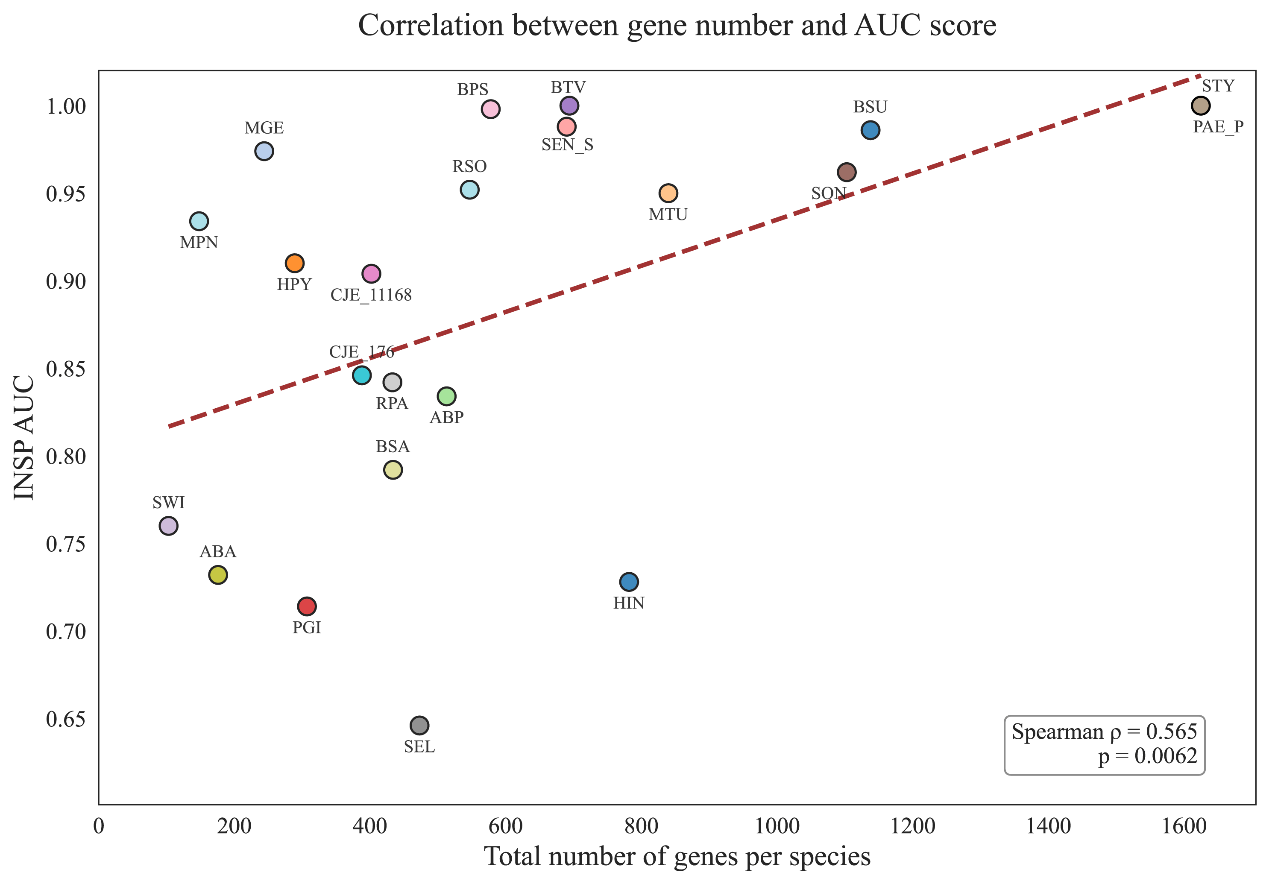


**Supplementary Figure S1** Scatter plot showing the relationship between the total number of genes used for training each species and the corresponding INSP AUC values across 22 bacterial species. Spearman’s rank correlation coefficient and the associated p-value are indicated.
