# Supplementary material for: CNN4Essential: a convolutional neural network model for predicting bacterial gene essentiality based on multi-feature fusion
Source: BMC Genomics. 2026 Apr 6;27:496. doi: 10.1186/s12864-026-12819-3 (PMC13188266; doi:10.1186/s12864-026-12819-3)
Supplement: Supplementary file 3 — Supplementary Material 3. [file 12864_2026_12819_MOESM3_ESM.docx]

**Supplementary Table S1** RefSeq accession numbers of the 22 bacterial species.

| **No.** | **Species** | **RefSeq Accession Number** |
| --- | --- | --- |
| 1 | *Bacillus subtilis* 168 | NC_000964 |
| 2 | *Haemophilus influenzae* Rd KW20 | NC_000907 |
| 3 | *Mycoplasma genitalium* G37 | NC_000908 |
| 4 | *Helicobacter pylori* 26695 | NC_000915 |
| 5 | *Mycobacterium tuberculosis* H37Rv | NC_000962 |
| 6 | *Salmonella typhimurium* LT2 | NC_003197 |
| 7 | *Acinetobacter baylyi* ADP1 | NC_005966 |
| 8 | *Porphyromonas gingivalis* ATCC 33277 | NC_010729 |
| 9 | *Bacteroides thetaiotaomicron* VPI-5482 | NC_004663 |
| 10 | *Salmonella enterica* subsp. Enterica serovar Typhimurium str. 14028S | NC_016856 |
| 11 | *Sphingomonas wittichii* RW1 | NC_009511 |
| 12 | *Shewanella oneidensis* MR-1 | NC_004347 |
| 13 | *Pseudomonas aeruginosa* PAO1 | NC_002516 |
| 14 | *Campylobacter jejuni* subsp. Jejuni NCTC 11168=ATCC 700819 | NC_002163 |
| 15 | *Burkholderia pseudomallei* K96243 | NC_006350, NC_006351 |
| 16 | *Synechococcus elongatus* PCC 7942 | NC_007604, NC_007595 |
| 17 | *Rhodopseudomonas palustris* CGA009 | NC_005296 |
| 18 | *Acinetobacter baumannii* ATCC 17978 | NC_009085 |
| 19 | *Brevundimonas subvibrioides* ATCC 15264 | NC_014375 |
| 20 | *Campylobacter jejuni* subsp. Jejuni 81-176 | NC_008787 |
| 21 | *Ralstonia solanacearum* GMI1000 | NC_003295.1, NC_003296.1 |
| 22 | *Mycoplasma pneumoniae* | NC_000912 |

The accession numbers were obtained from the NCBI RefSeq database and were used to retrieve complete genome sequences and corresponding gene annotations. These datasets served as the basis for sequence feature extraction and subsequent model training and evaluation.
